# Impact of a *Bacillus* Direct-Fed Microbial on Growth Performance, Intestinal Barrier Integrity, Necrotic Enteritis Lesions, and Ileal Microbiota in Broiler Chickens Using a Laboratory Challenge Model

**DOI:** 10.3389/fvets.2019.00108

**Published:** 2019-04-24

**Authors:** Daniel Hernandez-Patlan, Bruno Solis-Cruz, Karine Patrin Pontin, Xochitl Hernandez-Velasco, Ruben Merino-Guzman, Bishnu Adhikari, Raquel López-Arellano, Young Min Kwon, Billy M. Hargis, Margarita A. Arreguin-Nava, Guillermo Tellez-Isaias, Juan D. Latorre

**Affiliations:** ^1^Laboratorio 5: LEDEFAR, Unidad de Investigación Multidisciplinaria, Facultad de Estudios Superiores Cuautitlán, Universidad Nacional Autónoma de México, Cuautitlán Izcalli, Mexico; ^2^Departamento de Medicina Veterinária Preventiva, Centro de Diagnóstico e Pesquisa em Patologia Aviária, Universidade Federal do Rio Grande do Sul, Porto Alegre, Brazil; ^3^Departamento de Medicina y Zootecnia de Aves, Facultad de Medicina Veterinaria y Zootecnia, Universidad Nacional Autónoma de México, Ciudad de México, Mexico; ^4^Department of Poultry Science, University of Arkansas, Fayetteville, AR, United States; ^5^Eco-Bio LLC, Fayetteville, AR, United States

**Keywords:** *Bacillus*, broiler chickens, direct-fed microbial, ileal microbiota, necrotic enteritis

## Abstract

Decreases in the use of antibiotics and anticoccidials in the poultry industry have risen the appearance of necrotic enteritis (NE). The purpose of this study was to evaluate the effect of a *Bacillus* direct-fed microbial (DFM) on growth performance, intestinal integrity, NE lesions and ileal microbiota using a previously established NE-challenged model. At day-of-hatch, chicks were randomly assigned to three different groups: Negative control (NC), Positive control (PC) challenged with *Salmonella* Typhimurium (day 1), *Eimeria maxima* (EM, day 13) and *Clostridium perfringens* (CP, day 18–19), and *Bacillus*-DFM group (DFM) challenged as the PC. Body weight (BW) and body weight gain (BWG) were measured weekly. Total feed intake (FI) and feed conversion ratio (FCR) were evaluated at day 21. Liver samples were collected to assess bacterial translocation and blood samples were used to measure superoxide dismutase (SOD) and fluorescein isothiocyanate-dextran (FITC-d). Intestinal contents were obtained for determination of total IgA and microbiota analysis. NE lesion scores (LS) were performed at day 21. Chickens consuming the DFM significantly improved BW and had a numerically more efficient FCR compared to PC at day 21. Additionally, there were no significant differences in FCR between the DFM group and NC. Furthermore, the DFM group showed significant reductions in LS, IgA and FITC-d levels compared to the PC. However, there were no significant differences in SOD between the groups. The microbiota analysis indicated that the phylum *Proteobacteria* was significantly reduced in the DFM group in comparison to PC. At the genus level, *Clostridium, Turicibacter, Enterococcus*, and *Streptococcus* were reduced, whereas, *Lactobacillus* and *Bacillus* were increased in the DFM group as compared to PC (*p* < 0.05). Likewise, the DFM significantly reduced CP as compared to PC. In contrary, no significant differences were observed in bacterial composition between NC vs. DFM. In addition, beta diversity showed significant differences in the microbial community structure between NC vs. PC, and PC vs. DFM. These results suggest that the dietary inclusion of a selected DFM could mitigate the complex negative impacts caused by NE possibly through mechanism(s) that might involve modulation of the gut microbiota.

## Introduction

Necrotic enteritis (NE) is a multi-factorial pathology with significant financial influence on the poultry production (1). *Clostridium perfringens* (CP) type G previously identified as type A or C is a Gram-positive anaerobe-spore forming bacteria recognized as the primary etiology of NE. However, it is not well-known if the presence of other microorganisms contributes to disease onset or progression (2, 3). Pathogenic CP strains produce toxins, such as the α-toxin and/or the NetB-toxin associated with the pathology and increase intestinal permeability (4, 5). The alternatives to the use of antibiotics growth promoters (AGP) have also upsurge to support animal health and an efficient livestock production (6). Alternatives to control NE include direct-fed microbials (DFM) or probiotics, prebiotics, organic acids, phytochemicals, enzymes, and novel vector vaccines (7). Among the alternatives, probiotics have become a strong option because they are considered safe, do not pollute and have no residual compounds (8).

Bacteria from the genera *Bacillus* and *Lactobacillus* are probiotics that are intended to modify the gastrointestinal microflora generating advantageous activities to the host (9, 10). The modes of action of direct fed microbial (DFM) include: increase the population of beneficial bacteria (*Lactobacillus* and *Bifidobacterium* spp.), decrease pathogenic bacteria by competitive exclusion or the production of bacteriocins, stimulate metabolism by increasing endogenous and exogenous digestive enzyme activity, improve feed intake and digestion, reduce ammonia production, neutralize enterotoxins, enhanced the immune system status as well as the production of beneficial bacterial fermentation compounds, such as volatile fatty acids (7, 11).

Previously we have reported that inclusion of a *Bacillus* direct-fed microbial (DFM) candidate, selected based on different *in vitro* enzyme production profiles, was able to reduce digesta viscosity and CP proliferation in different diets using various cereal grains (12). However, the effect of the supplementation of the *Bacillus*-DFM candidate has not been evaluated *in vivo* in an established NE challenge model until now. Therefore, the present study was conducted to evaluate the effect of the dietary supplementation of the DFM on growth performance, NE lesion score (LS), bacterial translocation (BT), superoxide dismutase (SOD) activity, serum fluorescein isothiocyanate–dextran (FITC-d) concentration, total intestinal immunoglobulin A (IgA) levels and ileal microbiota of broiler chickens in a NE laboratory challenged model.

## Materials and Methods

### Necrotic Enteritis Model: Challenge Organisms

#### Salmonella Typhimurium

The poultry isolate of *Salmonella* Typhimurium (ST) was obtained from the USDA National Veterinary Services Laboratory (Ames, IA, United States). The ST isolate was selected for resistance to novobiocin (NO, 20 μg/mL, Sigma-Aldrich, St Louis, MO, USA) and nalidixic acid (NA, 25 μg/mL, catalog no. N4382, Sigma) in our laboratory. In the present study, 100 μL of ST from a frozen aliquot was added to 10 mL of tryptic soy broth (TSB, Sigma-Aldrich, St Louis, MO, USA) and incubated at 37°C, and passed three times every 8 h to ensure that all bacteria were in log phase as previously described (13). Post-incubation, bacterial cells were washed three times with sterile 0.9% saline by centrifugation at 1,864 × *g* for 10 min, reconstituted in saline, quantified by densitometry with a spectrophotometer (Spectronic 20D+, Spectronic Instruments Thermo Scientific, Rochester, NY), and diluted to an approximate concentration of 4 × 10^8^ cfu/mL. Then, 1 d-old broiler chickens were weighed and challenged with a concentration of 1 × 10^8^ cfu of ST per bird. The concentration of ST was further verified by serial dilution and plated on brilliant green agar (BGA, Sigma-Aldrich, St Louis, MO, USA) with NO and NA for enumeration of actual cfu used in the experiment.

#### Eimeria Maxima

Oocysts of the *Eimeria maxima*-M6 strain (EM) were donated by Dr. John. R. Barta, University of Guelph, Canada. The EM-M6 strain is a single sporocyst-derived isolate of a *E. maxima* strain isolated from litter samples in Florida, USA in 1994 and 1995 (14). *E. maxima* oocysts were propagated *in vivo* according to previously published methods (15). A preliminary dose titration study was carried out, to determine the EM challenge dose used in the present experiment (Data not shown). For the present study, the broiler chickens were weighed and challenged at 13 days of age by oral gavage with a dose of 2 × 10^4^ sporulated oocysts of EM per bird.

#### Clostridium Perfringens

Two different CP isolates were used in the present study. Dr. Jack L. McReynolds, USDA-ARS, College Station, TX, USA, kindly donated a CP strain that has been previously described in a NE challenge model and confirmed alpha-toxin positive (16). A frozen aliquot was amplified in TSB with sodium thioglycolate (Sigma-Aldrich, St Louis, MO, USA). The broth culture was plated on phenylethyl alcohol agar (PEA, Becton Dickinson, Sparks, MD, USA) with 5% sheep blood (Remel, Lenexa, KS, USA) to confirm purity; aliquots were made with 25% sterile glycerol and stored at −80°C until further use. The second CP strain was donated by Dr. Lisa Bielke, Ohio State University, USA and confirmed alpha-toxin and NetB positive. A single aliquot of each isolate was individually amplified overnight and plated on tryptic soy agar (TSA, Becton Dickinson, Sparks, MD, USA) with thioglycolate (THIO) to obtained the actual cfu number used during the challenge in the NE model. A mixed CP culture containing both isolates was administered on days 18 and 19 of age at a concentration of 1 × 10^9^ cfu per bird.

### Direct-Fed Microbials (DFM) Preparation

DFM preparation was performed as described earlier (17–19). The final product contained a concentration of stable *Bacillus* spores (~3 × 10^11^ spores/g). The DFM was mixed into the feed for 15 min using a rotary mixer to get the experimental diet with a final concentration of 10^6^ spores/g of feed. Samples of feed containing the DFM were subjected to 100°C for 10 min to eliminate vegetative cells and validate the number of spores per gram of feed after inclusion and mixing steps. Following heat-treatment, 10-fold dilutions of the feed samples were plated on TSA, letting spores in the feed sample germinate to vegetative cells after incubation at 37°C for 24 h, hence representing the number of spores present per gram of feed.

### Experimental Design

In the present study, an experiment was conducted to know the effect of a DFM on a necrotic enteritis (NE) model previously described by our laboratory (20). One hundred and twenty day-of-hatch male broiler chickens Cobb 500 (Siloam Springs, AR) were randomly assigned to one of three different treatment groups with four replicates of 10 broilers each (*n* = 40 birds/group): Group 1, no-challenged control (Negative control group, NC); Group 2, challenged control (Positive control group, PC) challenged with *Salmonella* Typhimurium (ST, day 1), *Eimeria maxima* (EM, day 13), and *Clostridium perfringens* (CP, day 18–19) and Group 3: challenge as PC+ DFM at a concentration of 1 × 10^6^ spores/g of feed. The chicks were raised in floor pens (300 cm × 150 cm) for 21 days, with their respective diet and water *ad libitum* and maintained at an age-appropriate temperature during the experiment. Body weight (BW) and body weight gain (BWG) were evaluated weekly. Feed intake and feed conversion ratio (FCR) were obtained at 21-days of age. Serum concentration of fluorescein isothiocyanate–dextran (FITC-d) was measured at day 21 after the calculation of an appropriate dose that was given by oral gavage. One-hour post-FITC-d gavage, all chickens were humanely euthanized by CO_2_ inhalation, and blood samples were collected from the femoral vein and centrifuged (1,000 × *g* for 15 min) to separate the serum from the red blood cells for FITC-d and superoxide dismutase (SOD) determination. Further, the right half of the liver from 12 broilers was aseptically collected in sterile sample bags (Nasco, Fort Atkinson, WI, USA) to evaluate bacterial translocation (BT). Additionally, intestinal samples for total intestinal IgA level, as well as the content of an ileal segment for microbiota analysis were collected. Finally, ileal NE lesion score (LS, *n* = 25 chickens/group) was evaluated as previously described (21) where 0 = no lesions; 1 = thin-walled and friable intestines; 2 = focal necrosis, gas production and ulceration; 3 = extensive necrosis, hemorrhage and gas-filled intestines; and 4 = generalized necrosis typical of field case, marked hemorrhage. This study was carried out in accordance with the recommendations of Institutional Animal Care and Use Committee (IACUC) at the University of Arkansas, Fayetteville. The protocol #15006 was approved by the IACUC at the University of Arkansas, Fayetteville for this study. Starter feed was used in this experiment and was formulated to approximate the nutritional requirements of broiler chickens as recommended by the National Research Council (22) and adjusted to breeder's recommendations (23). No antibiotics, coccidiostats or enzymes were added to the feed (Table 1).

**Table 1:** Ingredient composition and nutrient content of a basal starter diet used in the experiment on as-is basis.

**Item**	**Corn soybean-based diet**
Ingredient	(g/kg)
Corn	574.5
Soybean meal	346.6
Poultry oil	34.5
Dicalcium phosphate	18.6
Calcium carbonate	9.9
Salt	3.8
DL-Methionine	3.3
L-Lysine HCL	3.1
Threonine	1.2
Choline chloride 60%	2.0
Vitamin premix^a^	1.0
Mineral premix^b^	1.0
Antioxidant^c^	0.5
**CALCULATED ANALYSIS**	
Metabolizable energy (MJ/kg)	12.7
Crude protein (g/kg)	221.5

a *Vitamin premix supplied per kg of diet: Retinol, 6 mg; cholecalciferol, 150 μg; dl-α-tocopherol, 67.5 mg; menadione, 9 mg; thiamine, 3 mg; riboflavin, 12 mg; pantothenic acid, 18 mg; niacin, 60 mg; pyridoxine, 5 mg; folic acid, 2 mg; biotin, 0.3 mg; cyanocobalamin, 0.4 mg*.

b *Mineral premix supplied per kg of diet: Mn, 120 mg; Zn, 100 mg; Fe, 120 mg; copper, 10 to 15 mg; iodine, 0.7 mg; selenium, 0.2 mg; and cobalt, 0.2 mg*.

c *Ethoxyquin*.

### Bacterial Translocation

Briefly, liver samples were homogenized, weighed and 1:4 w/v dilution was made with sterile 0.9% saline with sodium thioglycolate. Then, 10-fold dilutions were plated on TSA with thioglycolate (catalog no. 212081, Becton Dickinson, Sparks, MD) for anaerobic bacteria (AB). Plates were then incubated anaerobically at 37°C for 24 h to enumerate total AB colony forming units. Simultaneously, liver samples were enriched in TSB with thioglycolate and further incubated at 37°C for 24 h. Following this, enrichment samples were confirmed negative or positive for AB by streak plating on TSA with thioglycolate. Bacterial translocation was expressed in colony forming units (log_10_ cfu/g of tissue).

### Serum Determination of FITC-d Gut Leakage

Intestinal leakage of FITC-d (MW 3–5 kDa; Sigma-Aldrich Co., St. Louis, MO, USA) and the measurement of its serum concentration was determined since FITC-d is a marker of paracellular transport and mucosal barrier dysfunction (24–27). One hour before humanely euthanizing the chickens by CO2 inhalation, 20 broiler chickens from each group were given an oral gavage dose of 8.32 mg/kg FITC-d (28), and five broiler chickens per group were used as no FITC-d control. FITC-d concentration from diluted sera was measured at an excitation wavelength of 485 nm and an emission wavelength of 528 nm (Synergy HT, Multi-mode microplate reader, BioTek Instruments, Inc., VT, USA).

### Superoxide Dismutase Activity

SOD activity was measured in 12 serum samples per group using a commercial assay kit (item No. 706002, Cayman chemical company, Michigan, USA) following the manufacturer's instructions. The three types of SOD (Cu/Zn, Mn, and FeSOD) were determined in samples diluted 1:5. Samples were measured at 450 nm using an ELISA plate reader (Synergy HT, multi-mode microplate reader, BioTek Instruments, Inc., Winooski, VT, USA).

### Total Intestinal Immunoglobulin a Levels

Total IgA levels were determined in 12 gut rinse samples per group as previously described (29). An intestinal section of 5 cm from Meckel's diverticulum to the ileocecal junction was taken and rinsed three times with 5 mL of 0.9% saline; then the rinse was collected in a tube and centrifuged at 1,864 × *g* at 4°C for 10 min. The supernatant was poured into a 96-microwell plate and stored at −20°C until tested. A commercial indirect ELISA set was used to quantify IgA according to the manufacturer's instructions (Catalog No. E30-103, Bethyl Laboratories Inc., Montgomery, TX 77356) in samples diluted 1:100. High protein-binding capacity 96-well plates (Catalog No. 439454, Nunc MaxiSorp, Thermo Fisher Scientific, Rochester, NY) were used, and samples were measured at 450 nm using an ELISA plate reader (Synergy HT, multi-mode microplate reader, BioTek Instruments, Inc., Winooski, VT, USA). Total intestinal IgA levels obtained were multiplied by the dilution factor (100) to determine the amount of chicken IgA in the undiluted samples.

### Ileal Microbiota Analysis via Illumina MiSeq

Six ileal samples from each group (NC, PC, and DFM) were used for ileal microbiota analysis through 16S rRNA gene sequencing. Briefly, about 200 mg of ileal contents from each sample were used for genomic DNA extraction using QIAamp® fast DNA stool mini kit (Qiagen, Catalog no. 51604, Valencia, CA, USA) following manufacturer's instructions, with the additional incorporation of a bead beating step. For bead beating, pellets from each sample were resuspended in 1 mL inhibit Ex buffer provided in the kit and transferred to 2 mL microcentrifuge tubes with screw cap (Thermofisher Scientific, Catalog no. 3468) containing 0.25 mL of sterile 0.1 mm glass beads (BioSpec, Mfr no. 11079101, Bartlesville, OK, USA). Bead beating was performed using a Bead mill 24 (Fisher Scientific) for six cycles of run time 0.30 s and stopping time 0.11 s between cycles. V1-V3 region of 16S rRNA gene from each 10 ng genomic DNA samples was amplified by using unique barcoded universal primers as previously described (30). PCR was performed using Q5® High-Fidelity DNA Polymerase (NEB; New England Biolabs, Beverly, MA, USA) in a final volume of 50 μL following manufacturer's instructions. The PCR protocol included an initial denaturation at 98°C for 30 s followed by 30 cycles of exponential amplifications using denaturation at 98°C for 10 s, annealing at 58°C for 30 s, extension at 72°C for 30 s, and the final extension at 72°C for 2 min. Amplicons were purified from 0.7% agarose gel, measured concentration using Qubit dsDNA broad range assay kit (Life Technologies, USA), and equal concentration (20 ng/μl) of amplicons were pooled together. The purified pooled amplicons were sequenced using MiSeq Illumina 300 cycle paired-end options at the University of California (Riverside, CA, USA).

### Data and Statistical Analysis

Data from anaerobic bacterial counts (AB Log_10_ cfu/g) in the liver, BW, BWG, FI, FCR, SOD activity, total IgA levels, serum FITC-d concentration, and NE lesion score were subjected to analysis of variance as a completely randomized design, using the General Linear Models procedure of SAS (31). Significant differences among the means were determined by Duncan's multiple range test at *p* < 0.05. Enrichment data were expressed as positive/total chickens (%), and the percent recovery of AB was compared using the chi-squared test of independence (32), testing all possible combinations to determine the significance (*p* ≤ 0.05).

Data from Illumina MiSeq sequencing was analyzed using Quantitative Insights into Microbial Ecology, QIIME version 1.9.1 [available at http://qiime.sourceforge.net/; (33)] at Jetstream cloud computing platform (34, 35). Paired-end reads were joined together using join_paired_ends.py command of QIIME with fastq-join option (36). After joining, barcode positions were formatted using customized Perl script and barcodes were removed using extract_barcodes.py command of QIIME. Split_libraries_fastq.py command of QIIME was used for demultiplexing and quality filtering of joined reads. Reads having Phred quality score of <20 were discarded. The chimeric sequences were identified using usearch61 [USEARCH version 6.1.544 (37)] and chimeric sequences along with shorter sequences (<100 bp) were excluded for downstream analysis. The OTU picking was performed using pick_open_reference_otus.py command of QIIME with usearch61 where a fraction of failure sequences to include in the subsample to cluster de novo was set at 0.1. The taxonomy was assigned based on green genes taxonomy and reference database version 13_8 (38) with the default method. For further statistical analysis and visual exploration, OTU table with taxa in plain format and metadata file were uploaded to the MicrobiomeAnalyst tool [available at http://www.microbiomeanalyst.ca; (39)]. Data were filtered using options: minimum count 2 and low count filter based on 20% prevalence in samples. Alpha diversity analysis was calculated based on the Shannon Index. Data were normalized using cumulative sum scaling before any statistical comparisons (40). Significant differences in alpha diversity among different groups were calculated based on the ANOVA/*T*-test where the significant difference level was set at *p* < 0.05. Beta diversity was calculated based on Weighted UniFrac distance metric, and statistical comparisons among groups were performed with Analysis of Similarities method (ANOSIM). To determine differentially abundant taxa at different groups, MetagenomeSeq (40) that uses zero-inflated Gaussian fit model was used with adjusted *P*-value cut off at 0.05.

## Results

### Overall Performance

In the present study, the effect of the dietary inclusion of DFM on growth performance of broiler chickens in the NE model is summarized in Table 2. During the first 7 days of age after the ST challenge, BW of PC was significantly reduced (11.66 g) as compared to NC, whereas in the DFM group, there were no significant differences when compared to PC and NC. Furthermore, similar BW values were observed between the group supplemented with DFM and NC at 14 day of age. However, in the third week, BW was significantly higher in the DFM group in comparison with PC and NC. BWG increased significantly by 33% in the second week (7–14 days), in broilers consuming the DFM compared to NC (*p* < 0.05).

**Table 2:** Evaluation of body weight (BW), body weight gain (BWG), feed intake (FI) and feed conversion ratio (FCR) in broiler chickens consuming a diet supplemented with or without DFM on a Necrotic enteritis challenge model^1^.

**Item**	**Negative control**	**Positive control**	**DFM**
**BW, G/BROILER**			
d 0	46.88 ± 0.64^b^	46.54 ± 0.64^b^	49.23 ± 0.68^a^
d 7	127.14 ± 2.90^a^	115.58 ± 3.27^b^	123.05 ± 3.80^ab^
d 14	273.80 ± 11.02^b^	295.78 ± 12.10^ab^	318.08 ± 13.57^a^
d 18	457.79 ± 18.97^ab^	456.32 ± 19.39^b^	525.58 ± 17.92^a^
d 21	603.81 ± 24.32^a^	445.96 ± 18.50^c^	507.77 ± 20.60^b^
**BWG, G/BROILER**			
d 0–7	80.39 ± 3.06^a^	67.74 ± 3.24^b^	75.08 ± 3.64^ab^
d 7–14	147.01 ± 9.51^b^	182.60 ± 9.48^a^	196.22 ± 10.56^a^
d 14–18	183.99 ± 9.85^ab^	160.55 ± 9.02^b^	198.31 ± 9.61^a^
d 14–21	325.78 ± 15.58^a^	152.13 ± 9.67^b^	185.27 ± 10.52^b^
d 0–21	552.72 ± 24.35^a^	399.42 ± 19.79^b^	458.58 ± 20.48^b^
**FI, G/BROILER**			
d 0–21	808.21 ± 29.86^a^	772.34 ± 10.66^a^	805.21 ± 71.07^a^
**FCR**			
d 0–21	1.46 ± 0.04^b^	1.93 ± 0.10^a^	1.76 ± 0.18^ab^

1 *Data expressed as mean ± SE from 40 chickens (4 replicates with 10 chicks each pen). P < 0.05*.

a−c *Values within columns with different superscripts differ significantly (p < 0.05)*.

Furthermore, the inclusion of the DFM prevented the BWG reduction seen in PC group after the EM challenge (14–18 days). Interestingly, there were no significant differences in BWG between the DFM and NC groups. At the end of the trial (21 days), a significant improvement in BW and a numerical increase in BWG were observed in the group supplemented with DFM as compared to PC. Feed intake (FI) was similar among groups at day 21 (Table 2). Feed conversion ratio (FCR) of PC (0–21 days) was significantly higher compared to the NC and 17 points less efficient compared to the DFM group (Table 2).

### Intestinal Integrity Parameters, Ileal Lesion Scores, Antioxidant Activity and IgA Production

The results of the dietary inclusion of the DFM on LS, BT, TSA/THIO, SOD activity, serum FITC-d concentration and total intestinal IgA levels in broiler chickens are shown in Table 3. In the case of LS, the DFM inclusion resulted in a significant decrease in the magnitude of intestinal damage observed in the ileum (From Meckel's diverticulum to the ileocecal junction) compared to PC group (Table 3). Additionally, broiler chickens consuming the DFM showed a numerical reduction in the number of AB translocated to the liver. No significant differences in SOD activity were found among the groups. Serum FITC-d concentration and intestinal IgA levels observed in the DFM group were similar to NC, and significantly lower (*p* < 0.05) than the challenged non-treated group (Table 3).

**Table 3:** Evaluation of ileal necrotic enteritis lesion scores (LS), bacterial translocation (BT) to the liver, superoxide dismutase (SOD) activity, serum concentration of fluorescein isothiocyanate–dextran (FITC-d) and immunoglobulin A (IgA) levels in broiler chickens.

**Treatments**	**LS^**1**^**	**BT Log_**10**_ cfu/g^**2**^**	**TSA/THIO ± (%)^**3**^**	**SOD (U/mL)^**4**^**	**FITC-d (μg/mL)^**5**^**	**IgA (μg/mL)^**6**^**
Negative control	0.33 ± 0.12^c^	1.52 ± 0.46^b^	6/12 (50%)	10.85 ± 0.55^a^	0.312 ± 0.048^b^	36.14 ± 3.79^b^
Positive control	2.04 ± 0.18^a^	3.34 ± 0.46^a^	10/12 (83%)	11.28 ± 0.59^a^	0.692 ± 0.050^a^	50.85 ± 4.48^a^
*Bacillus*-direct feed microbial	1.24 ± 0.18^b^	2.80 ± 0.50^ab^	9/12 (75%)	12.20 ± 0.26^a^	0.356 ± 0.046^b^	33.00 ± 2.75^b^

a−c *Values within treatment columns for each treatment with different superscripts differ significantly (p < 0.05)*.

1 *LS was evaluated in 25 broiler chickens*.

2 *Data expressed in Log_10_ cfu/g of tissue. Mean ± SE from 12 chickens. p < 0.05*.

3 *Data are presented as positive/total chickens (%)*.

4 *SOD activity from 12 serum samples*.

5 *FITC-d concentration from 20 serum samples*.

6 *IgA levels determined in 12 intestine samples*.

#### Analysis of Ileal Microbiotas

**Figure 1:**
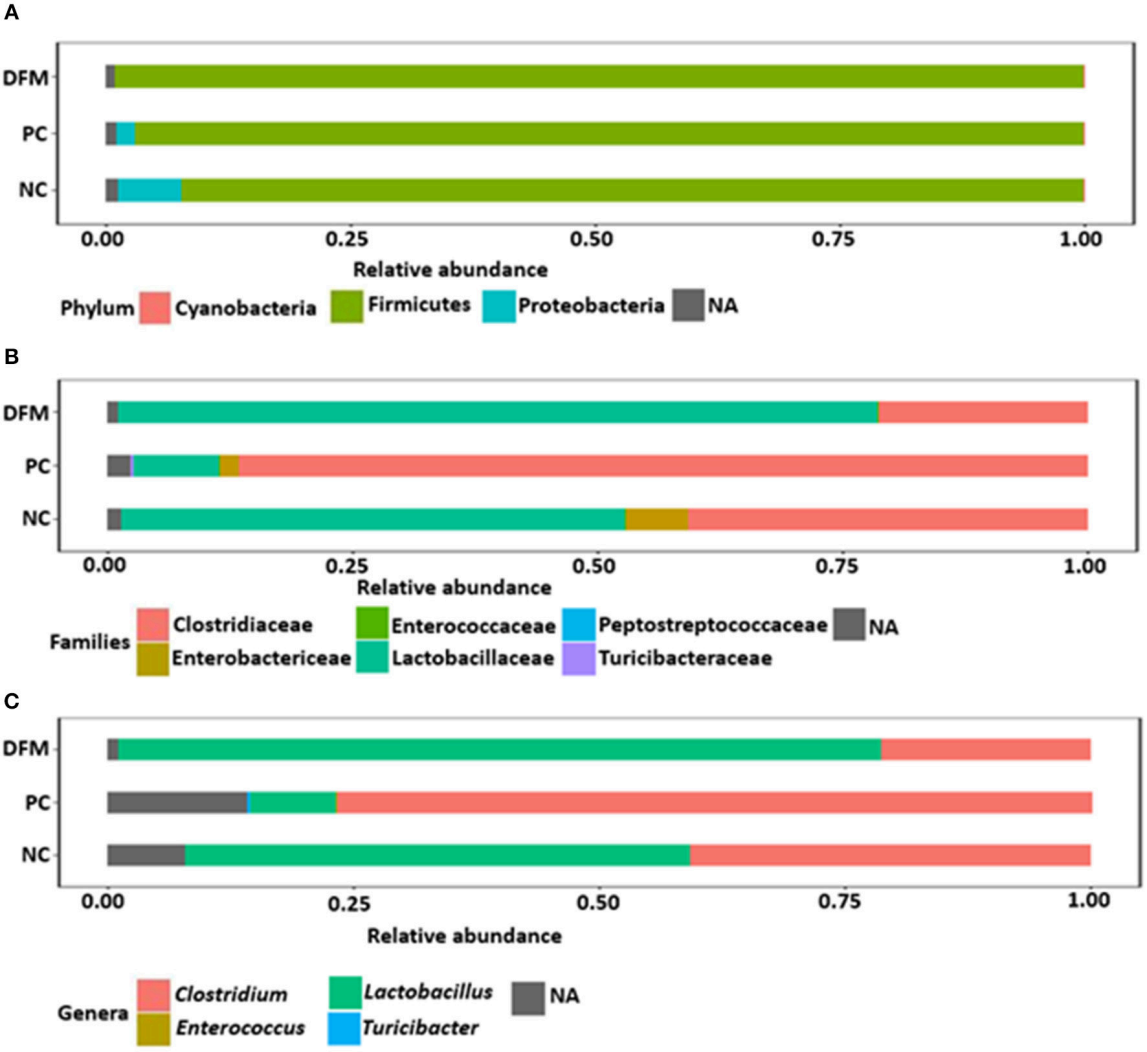
Relative abundance of different phyla **(A)**, families **(B)** and genera **(C)** in different treatment groups (NC, PC, and DFM). NA refers to those reads that were not assigned to the respective taxonomic levels.

After quality filtering and demultiplexing there were 914,899 reads with median sequence length of 500 bp. Summarizing of OTU table resulted in a mean sequence depth of 47,395 per sample. The OTU numbers in different samples varied from 100 to 642. The relative abundance of different bacterial phyla, families, and genera in NC, PC and the group of broiler chickens supplemented with DFM are shown in Figure 1. Taxonomic analysis revealed that Firmicutes was found as a predominant phylum which constituted >90% of total reads and *Proteobacteria* was reduced in the DFM group as compared to both PC and NC groups, though significant difference (*p* < 0.05) was observed only when compared to PC (Figure 1A and Table 4). At family level, *Clostridiaceae* is the most dominating bacterial family in PC group (86.53%), whereas Lactobacillaceae is the most dominating family in both NC (51.52%) and DFM (77.59%) groups (Figure 1B). Likewise, *Clostridium* was found the highest in PC group (76.94%), whereas *Lactobacillus* was reported highest in the DFM group (77.57%). For NC, *Clostridium* (40.74%) and *Lactobacillus* (51.60%) were found in between the PC and the DFM groups (Figure 1C).

**Table 4:** Differentially abundant bacterial taxa between PC and DFM group (MetagenomeSeq, *p* < 0.05).

**DFM**	**PC**
**PHYLUM LEVEL**	
	Proteobacteria
**FAMILY LEVEL**	
Lactobacillaceae Bacillaceae	Clostridiaceae Enterobacteriaceae Turicibacteraceae Enterococcaceae Streptococcaceae Peptostreptococcaceae
**GENUS LEVEL**	
*Lactobacillus* *Bacillus*	*Clostridium* *Turicibacter* *Enterococcus* *Streptococcus*
**SPECIES LEVEL**	
	*C. perfringens*

The differentially abundant taxa at different taxonomic levels between NC vs. PC and DFM vs. PC as identified by MetagenomeSeq (*p* < 0.05) were shown in Tables 4, 5, respectively. *Clostridiaceae*, Turicibacteraceae, and *Enterococcaceae* were significantly more abundant in PC as compared to NC. In contrast, Lactobacillaceae and *Enterobacteriaceae* were significantly abundant in NC as compared to PC (Table 5). Furthermore, when comparing NC vs. PC, *Clostridium, Turicibacter*, and *Enterococcus* were found significantly higher in PC, whereas *Lactobacillus* was found significantly higher in NC (Table 5).

**Table 5:** Differentially abundant bacterial taxa between NC and PC group (MetagenomeSeq, *p* < 0.05).

**NC**	**PC**
**FAMILY LEVEL**	
*Lactobacillaceae* *Enterobacteriaceae*	*Clostridiaceae* Turicibacteraceae *Enterococcaceae*
**GENUS LEVEL**	
*Lactobacillus*	*Clostridium* *Enterococcus* *Turicibacter*

Similarly, Lactobacillaceae and Bacillaceae were significantly higher in the DFM group as compared to PC, whereas *Clostridiaceae, Enterobacteriaceae*, Turicibacteraceae, *Enterococcaceae, Streptococcaceae*, and Preptostreptococcaceae were significantly higher in PC as compared to the DFM group (Table 4). Also, *Lactobacillus* and *Bacillus* were found significantly higher in the DFM group as compared to PC. However, *Clostridium, Turicibacter, Enterococcus*, and *Streptococcus* were significantly higher in PC group as compared to the DFM group (Table 4). In accordance with *Clostridium*, CP was also significantly reduced in the DFM group as compared to PC group (Table 4). Interestingly, there were no significant differences observed between NC vs. DFM at any taxonomic levels, suggesting that DFM may play a vital role to restore the gut dysbiosis occurred in the PC group.

**Figure 2:**
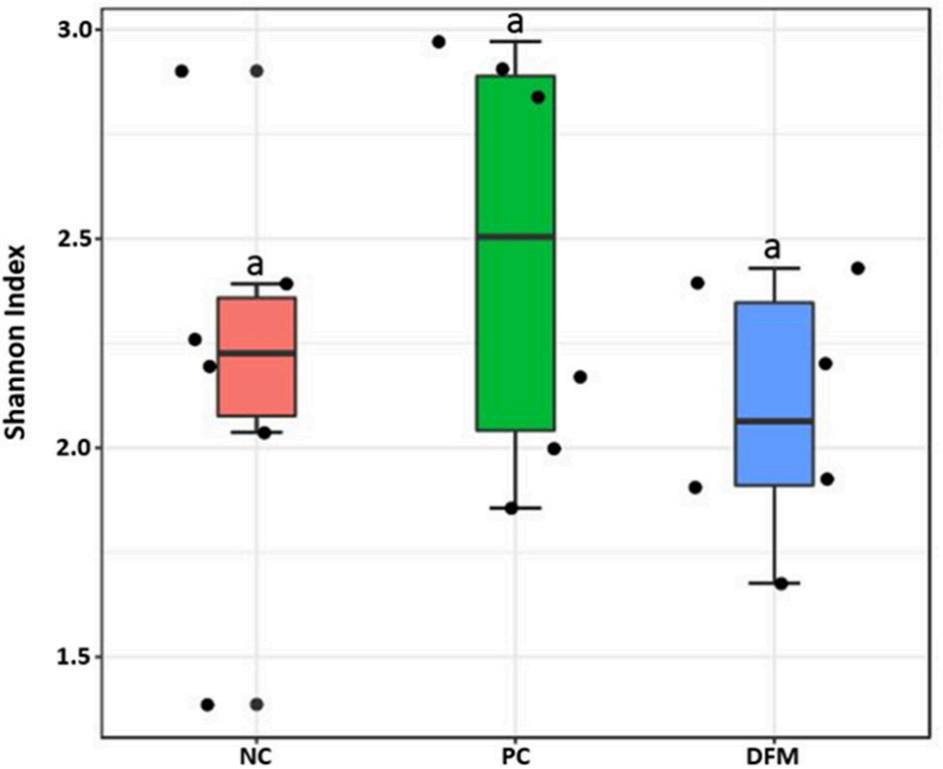
Alpha diversity of the three different groups (NC, PC, and the DFM group) as measured by Shannon Index. No significant difference was observed among three groups (ANOVA, *p* > 0.05).

Alpha diversity among the three groups as measured by Shannon Index is shown in Figure 2. Although the alpha diversity was higher in PC, there were no significant differences observed among the groups.

**Figure 3:**
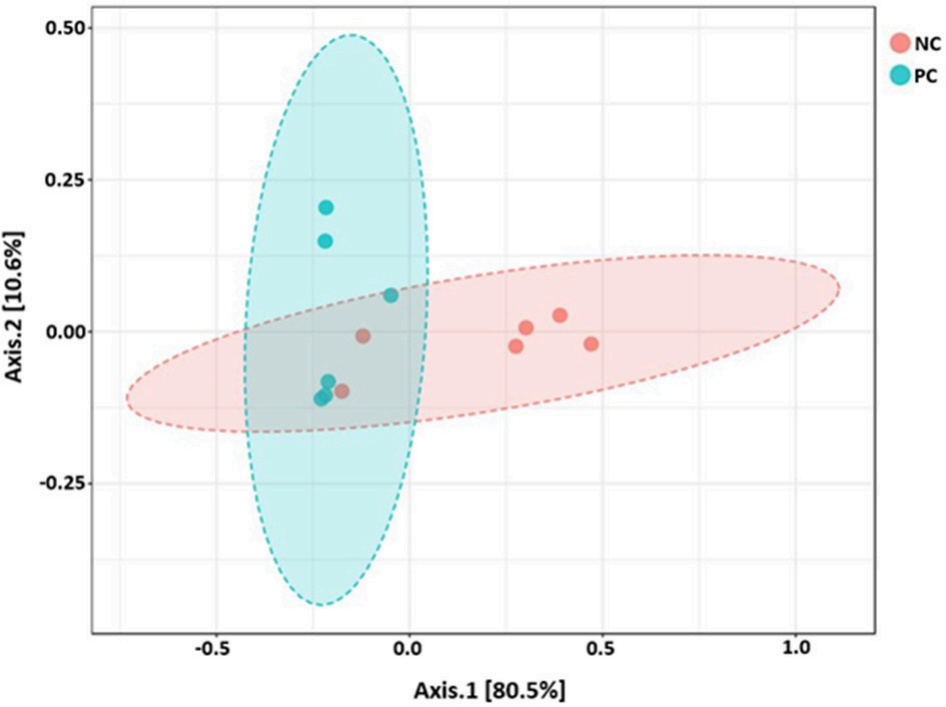
PCoA plot showing difference in microbial community structure between NC and PC (ANOSIM; *R* = 0.40 and *p* < 0.05).

**Figure 4:**
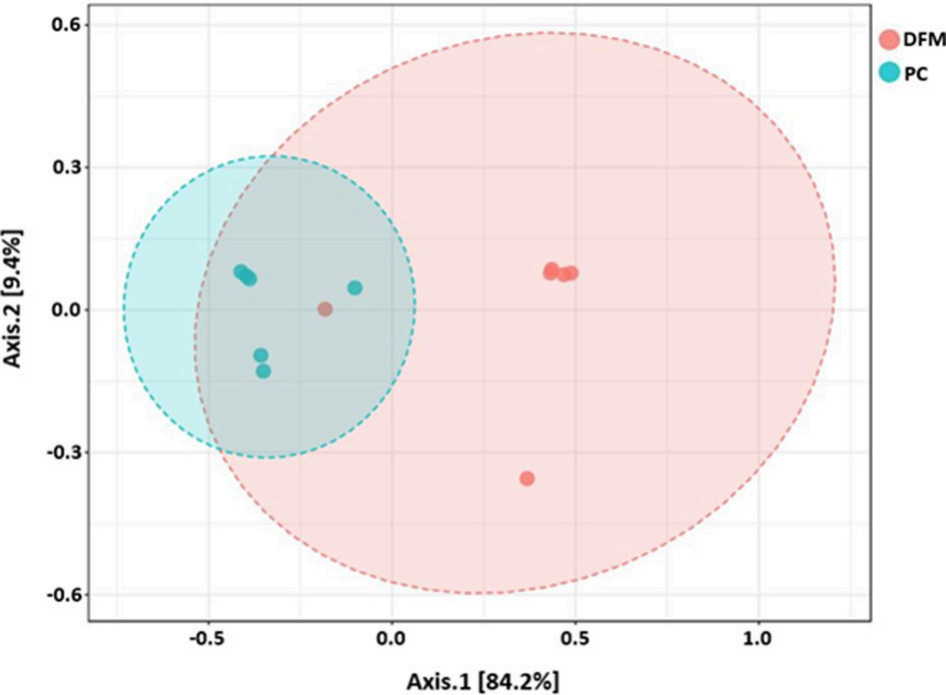
PCoA plot showing difference in microbial community structure between the DFM group and PC (ANOSIM; *R* = 0.73 and *p* < 0.01).

**Figure 5:**
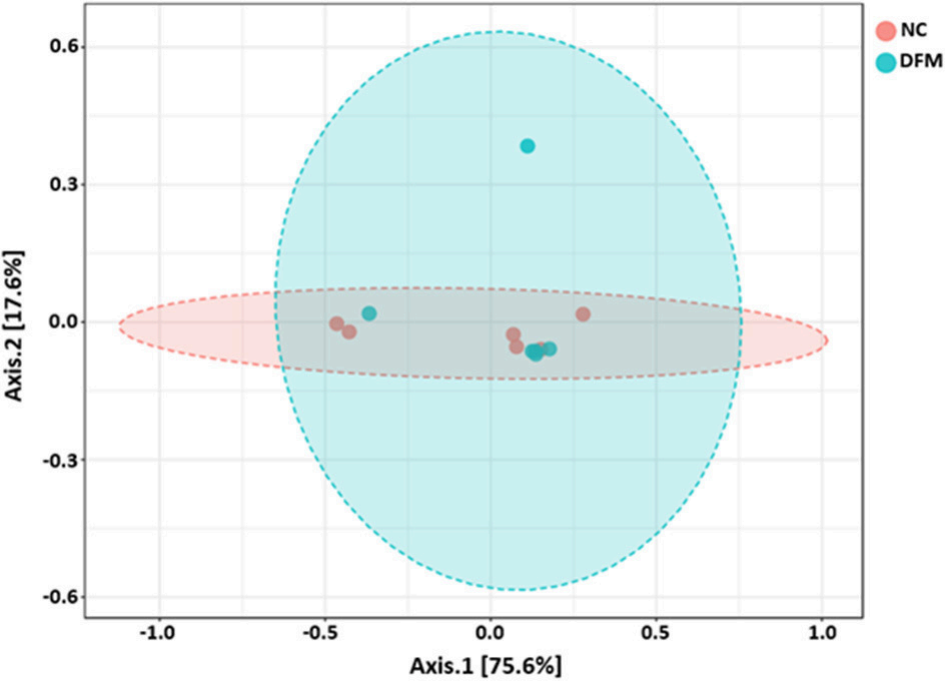
PCoA plot showing no difference in microbial community structure between the DFM group and NC (ANOSIM; *R* = −0.02 and *p* > 0.05).

Beta diversity between NC vs. PC, DFM vs. PC, and NC vs. DFM, as measured by the weighted UniFrac distance metric are illustrated in PCoA plots (Figures 3–**5**, respectively). Analysis of similarities (ANOSIM) result showed significant differences in microbial community structure between NC and PC groups (*R* = 0.40 and *p* < 0.05) and between the DFM and PC groups (*R* = 0.73 and *p* < 0.01). However, in agreement with above taxonomic results, there was no significant difference in microbial community structure between NC and DFM which furthermore supports the role of DFM in normalization of the gut dysbiosis caused by NE challenge model.

## Discussion

Several studies have shown that the use of live microorganisms as DFMs represents a potential alternative to antibiotics for the prevention and treatment of NE (41, 42). The present study was conducted to evaluate the effect of the dietary supplementation of the DFM on growth performance, ileal lesion scores, bacterial translocation, serum FITC-d concentration, SOD activity, total intestinal IgA and ileal microbiota of broiler chickens in a NE model. Growth performance of broiler chickens was significantly less efficient in PC compared to NC, whereas BW was significantly lower and FCR was 17 points higher in the PC when compared to the DFM group (Table 2). The reduction in BWG and impaired FCR is related to the intestinal damage derived from the ST, EM and CP challenges, since the absorption and utilization of nutrients are affected and, therefore, there is a reduction in the growth rate and a reduced feed conversion efficiency (20, 43, 44). In previous studies, the administration of this *Bacillus-*DFM was associated with significant reductions in supernatant viscosity and *C. perfringens* proliferation, suggesting that the exogenous enzymes produced by the DFM could decrease the amount of nutrients available for *C. perfringens* to grow, improving the intestinal microbial environment and enhancing the rate of passage of the digesta throughout the GIT (12, 45). Furthermore, we have demonstrated that the *Bacillus-*DFM showed antimicrobial activity against different food-borne pathogens, including *Salmonella* Enteritidis, *Escherichia coli*, and *Clostridium difficile* (45). Therefore, the administration of the DFM is providing multiple benefits related to the microbiological, chemical and physical features of the intestinal content. Consequently, the consumption of the *Bacillus*-DFM may contribute to enhanced performance through improving nutrient digestibility, maintaining a beneficial gut microbiota, and promoting a healthy intestinal integrity (12, 18, 44).

One of the most important predisposing factors in NE is the EM infection because it can induce mucogenesis and then provide a niche for CP colonization and proliferation, followed by the release of toxins and intestinal damage (46). Ileal NE lesion scores were significantly higher in PC compared to NC and DFM groups (Table 3). The damage of the intestinal mucosa caused by EM and CP induces strong inflammation responses, altering the intestinal permeability and favoring “leaky gut” (47). This change in permeability was proved with the significant increase in serum FITC-d concentration and BT in PC groups as compared to NC and DFM groups (Table 3). The reductions of the NE lesion score in the DFM group might be related to a combination of benefits including production of beneficial chemical compounds, immunoregulation of inflammation, and stimulation of intestinal microbiota homeostasis, resulting in an appropriate intestinal health status (48).

Production of intestinal IgA provides a critical mucosal immunity against microorganisms, as well as the inhibition of inflammatory processes and enhancement of non-specific defense mechanisms (29, 49). In the present study, PC group showed the highest intestinal IgA levels (*p* < 0.01) compared to NC and the DFM groups (Table 3). These results are as expected since PC was the group with the most severe intestinal damage, which induces an intense local inflammatory response, leading to an increase of pro-inflammatory cytokines and subsequently IgA production to promote epithelial repair (50, 51). The significant decrease in intestinal IgA levels in the DFM group could be related to the low impact of EM and CP challenge by reducing levels of inflammatory responses, given its anti-inflammatory effect in the intestinal epithelia (52).

Although previous *in vitro* studies have shown that the DFM reduce the concentration of CP (12), this is the first time that the intestinal microbiota has been analyzed. Much like for other vertebrates, Firmicutes, Bacteroidetes, and *Proteobacteria* are the predominant phyla (>90%) in the avian gut (53), which is also supported by the results from our study. Although no significant differences were found in the phylum Firmicutes, there was a significant decrease in *Proteobacteria* in the DFM group in comparison to PC (Figure 1A and Table 4). Interestingly, this *Bacillus-*DFM has previously shown antimicrobial activity against different food-borne pathogens including bacteria from the *Salmonella* spp. (45), and in the current study to reproduce NE an early challenge with *S*. Typhimurium was required as a crucial predisposing factor. It is possible that the antimicrobial compounds produce by DFM had an impact on reducing ST infection, and may be related to the difference in the relative abundance of *Proteobacteria* in comparison to the PC. On the other hand, consumption of the DFM did not change the abundance of bacteria from the phylum Firmicutes in comparison to the PC. Nevertheless, it modified the composition at the family and genus level of bacteria from the same phylum in both groups, increasing the abundance of known beneficial bacteria such a *Lactobacillus* and *Bacillus* in the DFM group in comparison to the PC. NE is an enteric disease which can disrupt the normal equilibrium of the intestinal microbes resulting in gut dysbiosis (54). It has been reported that NE severity is related to an increase in *Proteobacteria* and a decrease in Firmicutes phyla (8) since Firmicutes population is important to suppress or eliminate CP and restore the intestinal homeostasis (55). Although under healthy conditions, CP is always found at levels lower than 10^5^ cfu/g of intestinal content (41). Therefore, it is probable that the DFM alleviates the gut dysbiosis toward a normal intestinal microbiota. On the other hand, the *Proteobacteria* population in NC was higher compared to PC and DFM groups because a single sample had higher abundance value, causing an increase in the presence of this phylum (Figure 1A). However, Tables 4, 5 clearly showed that there was no significant difference in the abundance of bacterial taxa between NC vs. DFM, contrary to NC vs. PC and PC vs. DFM. Furthermore, the genus *Clostridium* was significantly higher in PC compared to NC and DFM groups because of the changes in the intestinal microflora derived from NE (56) (Figure 1C).

In contrast, the genus *Lactobacillus* was significantly predominant in both NC and DFM groups with respect to PC, it was even higher in the DFM group than in NC group (Figure 1B). The genus *Lactobacillus* plays a crucial role in the homeostasis of the gastrointestinal tract of metazoans (9). The DFM was able to increase the genus *Lactobacillus* (Figure 1C) probably because it prevented dysbiosis and maintains gut integrity (41).

The genera *Turicibacter, Streptococcus, Enterococcus*, and *Clostridium* were significantly higher in PC group due to the change in the ileum microbiota caused by NE. The genera *Lactobacillus* and *Bacillus* were higher in the group receiving DFM, suggesting that these genera might be playing a vital role in alleviating the negative impacts caused by CP (57). MetagenomeSeq analysis showed that the genus *Clostridium* was significantly more abundant in PC (Table 5) when compared to NC (Table 4). In addition, both *Clostridium* and CP were significantly reduced by DFM as compared to PC (Table 4), providing again evidence that NE was less severe in this group. However, there were no significant differences in any bacterial taxa between NC and DFM suggesting the possible role of DFM in restoration of gut dysbiosis occurred in PC group.

Finally, there were no significant differences in bacterial alpha diversity among the groups (Figure 2), because the NE model used was designed to produce low mortality since, in other studies it has been proved that only extreme events can modify the alpha diversity (58). In contrast, significant differences in beta diversity were found when comparing PC with either NC or DFM groups (Figures 3, 4), which agrees with another study, in which it was demonstrated that NE cause significant changes in the intestinal microbiota (59). Interestingly, there was no difference in bacterial community structure between NC and DFM as demonstrated in PCoA plot (Figure 5), which further confirms that DFM played a vital role in restoring the gut dysbiosis in this study.

The results of this study suggest that the dietary inclusion of a selected DFM could diminish the undesirable effects of NE on leaky gut and reduce inflammation, hence improve growth performance. The improvement in intestinal health by DFM as indicated by the decrease in serum FITC-d concentration, IgA, LS, and BT may be achieved through mechanism(s) that might involve the modulation of gut microbiota. Because DFM had significantly reduced the abundance of CP, while increased the abundance of *Lactobacillus* and *Bacillus* suggesting that these genera might play a vital role in suppressing CP and reducing the detrimental effects of NE. In addition, DFM restored gut microbiota as comparable to the NC which further confirms the role of DFM in modulating gut microbiota to reduce the effects of NE. Besides these, the decrease in CP could also have been due to the ability of the DFM to produce antimicrobial compounds, degradation of CP toxins by synthesis of exogenous enzymes and/or increasing the expression of tight junction proteins which improve the intestinal barrier integrity. Thus, our present study provides new insights into the prevention and treatment of NE in broiler chickens. Nevertheless, consideration of the probable synergistic effects of a blend of DFM with other feed additives may be necessary for future NE studies.

## Ethics Statement

All animal handling procedures complied with the Institutional Animal Care and Use Committee (IACUC) at the University of Arkansas, Fayetteville (protocol #15006).

## Author Contributions

DH-P, BS-C, BA, and GT-I contributed to the overall study design and supervised all research. KP, JL, BA carried out the experiments, DH-P, GT-I, XH-V, RM-G, MA-N, and BS-C analyzed the data and drafted and revised the first version of the manuscript. DH-P and BS-C prepared figures. BA, BS-C, YK, BH, RL-A, MA-N, and GT-I contributed partly to writing and finally revising the manuscript and data analysis, and were also responsible for the final editing of the manuscript. All the authors reviewed, edited, and approved the manuscript.

### Conflict of Interest Statement

MA-N was employed by Eco-Bio LLC. The remaining authors declare that the research was conducted in the absence of any commercial or financial relationships that could be construed as a potential conflict of interest.
